# QTL Detection for Kernel Size and Weight in Bread Wheat (*Triticum aestivum* L.) Using a High-Density SNP and SSR-Based Linkage Map

**DOI:** 10.3389/fpls.2018.01484

**Published:** 2018-10-11

**Authors:** Qiannan Su, Xilan Zhang, Wei Zhang, Na Zhang, Liqiang Song, Lei Liu, Xin Xue, Guotao Liu, Jiajia Liu, Deyuan Meng, Liya Zhi, Jun Ji, Xueqiang Zhao, Chunling Yang, Yiping Tong, Zhiyong Liu, Junming Li

**Affiliations:** ^1^Center for Agricultural Resources Research, Institute of Genetics and Developmental Biology, Chinese Academy of Sciences, Shijiazhuang, China; ^2^The College of Life Science, University of Chinese Academy of Sciences, Beijing, China; ^3^College of Biology and Engineering, Hebei University of Economics and Business, Shijiazhuang, China; ^4^State Key Laboratory of Plant Cell and Chromosome Engineering, Chinese Academy of Sciences, Beijing, China; ^5^Anyang Academy of Agricultural Sciences, Anyang, China

**Keywords:** linkage analysis, SNP, SSR, QTL, kernel traits, *Triticum aestivum*

## Abstract

High-density genetic linkage maps are essential for precise mapping quantitative trait loci (QTL) in wheat (*Triticum aestivum* L.). In this study, a high-density genetic linkage map consisted of 6312 SNP and SSR markers was developed to identify QTL controlling kernel size and weight, based on a recombinant inbred line (RIL) population derived from the cross of Shixin828 and Kenong2007. Seventy-eight putative QTL for kernel length (KL), kernel width (KW), kernel diameter ratio (KDR), and thousand kernel weight (TKW) were detected over eight environments by inclusive composite interval mapping (ICIM). Of these, six stable QTL were identified in more than four environments, including two for KL (*qKL-2D* and *qKL-6B.2*), one for KW (*qKW-2D.1*), one for KDR (*qKDR-2D.1*) and two for TKW (*qTKW-5A* and *qTKW-5B.2*). Unconditional and multivariable conditional QTL mapping for TKW with respect to TKW component (TKWC) revealed that kernel dimensions played an important role in regulating the kernel weight. Seven QTL-rich genetic regions including seventeen QTL were found on chromosomes 1A (2), 2D, 3A, 4B and 5B (2) exhibiting pleiotropic effects. In particular, clusters on chromosomes 2D and 5B possessing significant QTL for kernel-related traits were highlighted. Markers tightly linked to these QTL or clusters will eventually facilitate further studies for fine mapping, candidate gene discovery and marker-assisted selection (MAS) in wheat breeding.

## Introduction

Bread wheat (*Triticum aestivum* L.) is one of the leading cereal crops worldwide, which plays a crucial role in sustaining food security. The genetic improvement of three yield components, i.e., productive spikes per unit area, kernel number per spike and kernel weight, contributed a great deal to the increase of wheat yield level and alleviation of food crisis in the last decades (Sayre et al., 1997). Among the three yield components, kernel weight showed the highest heritability (Alexander et al., 1984), and selection for this component in the early generations of breeding was highly effective (Xiao and He, 2003; Brown et al., 2009). For instance, thousand kernel weight (TKW) of Chinese wheat mini core collection (262 accessions) increased from a mean 31.5 g in 1940s to 44.64 g in 2000s, representing a 2.19 g increase in each decade (Wang L. et al., 2012).

Thousand kernel weight is closely associated with kernel size traits, such as kernel length (KL), kernel width (KW), and kernel diameter ratio (KDR) (Campbell et al., 1999; Dholakia et al., 2003). And kernel size traits usually contribute to yield by affecting the TKW and can also be associated with milling and processing (Osborne and Anderssen, 2003). Therefore, improving kernel weight and size is a prime breeding target for wheat yield potential and end use quality.

Thousand kernel weight and kernel size are complex quantitative traits controlled by multiple genes and significantly influenced by the environment (Giura and Saulescu, 1996; Ammiraju et al., 2001). Quantitative trait loci (QTL) mapping is a key approach to understand the genetic architecture of kernel traits. Great progress has been made in identifying major QTL and isolating underlying genes for kernel weight and size in cereal crops, such as rice, maize (Li et al., 2010) and barley (Ayoub et al., 2002), etc. For rice, in particular, lots of genes controlling the kernel size and weight have been characterized, like *GS3* (Fan et al., 2006), *GS5* (Li et al., 2011), *qGL3* (Zhang X. et al., 2012), *GW2* (Song et al., 2007), and *GW8* (Wang S. et al., 2012). Recently, Xu et al. (2018) reported that the OsMKKK10-OsMKK4-OsMAPK6 signaling pathway positively regulates grain weight and size in rice. Results of these studies revealed that grain yield was controlled by genes related to kernel size traits.

Compared with rice, the molecular cloning of genes associated with kernel weight and size has lagged behind in wheat. Up to date, none gene/QTL associated with kernel weight and size have been cloned in wheat via the map-based cloning approach. And most of them, such as *TaCwi-A1* (Ma et al., 2012), *TaCKX6-D1* (Zhang L. et al., 2012), *TaGS1* (Guo et al., 2013), *TaGS5* (Ma et al., 2016), and *TaGW2* (Su et al., 2011) were cloned through homologous cloning. Although multiple major and stable QTL controlling kernel shape and size were identified on chromosomes 2A (Wu et al., 2015), 2D (Breseghello and Sorrells, 2007; Ramya et al., 2010; Cui et al., 2016), 4A (Cui et al., 2016), 5A (Wu et al., 2015), 5B (Breseghello and Sorrells, 2007; Ramya et al., 2010; Li et al., 2015) and 6A (Zhang et al., 2013), they were defined in a relatively large chromosome interval due to the limited numbers of markers.

Single nucleotide polymorphism (SNP) is the most abundant type of molecular markers. During the past 5 years, increasing numbers of SNPs have been discovered in hexaploid wheat. For example, Cavanagh et al. (2013) released a consensus genetic map with 7504 SNP markers from the Wheat9K SNP array using a combination of seven mapping populations. Wang et al. (2014) mapped 46,977 SNP markers from the Wheat90K array to the wheat genetic map using a combination of eight mapping populations. Winfield et al. (2016) documented a consensus map with 56,505 SNP markers from the Wheat820K array, using three independent bi-parental populations. Recently, using Wheat660K array, Cui et al. (2017) released a high-density genetic map with 119,566 markers (including 119,001 SNP markers) based on an individual mapping population. Using the high-throughput SNP genotyping, more and more wheat QTL for kernel traits have been high-solution mapped.

In the present study, a high-density genetic linkage map based on iSelect 90K SNP and SSR markers was constructed using the Shixin828/Kenong2007 recombinant inbred line (RIL) population. Both unconditional and conditional QTL analysis were conducted to investigate the underlying genetic basis of TKW and kernel size as well as to dissect the genetic relationships between them at QTL level. The information obtained from this study could provide further insights into the genetic factors that influence kernel traits.

## Materials and Methods

### Plant Materials and Field Trials

An F_6:7_ RIL population derived from a cross between Shixin828 (SX828) and Kenong2007 (KN2007) (denoted by SK-RIL) was developed in this study. SX828 was released in 2005 and has been one of the major cultivars in the North China Plain in the last decade. It has superior photosynthesis characteristics during filling stage (Bi et al., 2010), thus shows higher grain-filling rate and larger kernel than most other commercial cultivars. KN2007, on the other hand, is a small kernel line derived from Kenong9204 (Cui et al., 2011). In the present study, 163 SK-RILs were used for SNP and SSR genotyping, genetic linkage analysis and QTL detection.

The SK-RILs and their parents were evaluated in eight environments: 2014–2015 in Shijiazhuang (37°53′N, 114°41′E, altitude 54 m) with high nitrogen (HN) trial; 2015–2016 in Shijiazhuang with both HN and low nitrogen (LN) trials; 2016–2017 in Shijiazhuang with both HN and LN trials, Anyang (35°12′N, 113°37′E, altitude 77 m) with HN trial, Beijing (40°06′N, 116°24′E, altitude 51 m) with both HN and LN trials. These 8 environments were designated as E1, E2, E3, E4, E5, E6, E7, and E8, respectively. The soil nitrate-nitrogen (N) contents within the 0–20 cm layer in each environment were measured after harvest (**Supplementary Table S1**). In each HN plot, 300 kg ha^-1^ of diamine phosphate and 225 kg ha^-1^ of urea were applied before sowing, and 150 kg ha^-1^ of urea was applied at the elongation stage every year. In the LN plots, no nitrogen fertilizer was applied throughout the growing period. The materials were planted in randomized complete blocks with two replications for each of the 8 environments. Each block contained two rows that were 2 m long and 0.25 m apart and 40 seeds were evenly planted in each row. All of the recommended agronomic practices were followed in each of the trials except for the nitrogen fertilization treatment described above.

### Phenotypic Evaluation and Statistical Analysis

Five representative plants in the center of the second row were randomly sampled at physiological maturity for phenotypic evaluation. Kernel traits including KL, KW, KDR, and TKW were evaluated for 200 random kernels from all tiller spikes of each representative plants using the Seed Counting and Analysis System of WSeen SC-G Instrument (Zhejiang, China)^1^. The spike number per plant (SNPP) and kernel number per spike (KNPS) were also investigated in the 8 environments detailed above. SNPP was determined by the mean of the five representative plants, while KNPS was determined by the mean of the main spikes of the five representative plants.

The analysis of variance (ANOVA) and the calculation of phenotypic data correlation coefficients between all investigated traits were performed with SPSS 20.0 (SPSS, Chicago, IL, United States)^2^. Broad sense heritability ($h_{B}^{2}$) of the corresponding traits was estimated based on the following formula: $h_{B}^{2}$ = *V*_G_/*V*_P_; where *V*_G_ and *V*_P_ are the genetic variance and phenotypic variance, respectively. The conditional phenotypic values of TKW with respect to TKW component (TKWC) were evaluated using QGAStation 2.0^3^ according to Zhu (1995) and Fan et al. (2015). The raw data from each environment were assembled as follows: the first column represented the block (replications), the second column represented the genotype (163 SK-RILs), and the following columns were trait data, specifically the TKWC and TKW. ‘Conditional Final’ was conducted and the output file provided information on conditional phenotypic values of y_(TKW|TKWC)_, which indicates TKW conditioned on TKWC (for example, TKW| KL means TKW was conditioned on KL). Both unconditional and conditional phenotypic values were used for QTL mapping analyses.

### Genotyping and Linkage Map Construction

The 163 SK-RILs together with their two parents were genotyped using the Illumina iSelect 90K SNP Array containing 81,587 wheat SNP markers from CapitalBio Corporation (Beijing, China)^4^. In addition, 225 SSR markers were used to anchor the linkage groups into specific chromosomes better. Marker allele frequency < 0.3 or containing > 10% missing data were rejected. The remaining markers were binned based on the pattern of segregation in the SK-RILs using the BIN function of IciMapping 4.1.^5^ Unique markers from each bin with least missing data were further identified and sorted into groups using the MAP function of IciMapping 4.1, with a LOD score of 3.5 and a recombination fraction of 0.3 using the Kosambi mapping function. Groups were ordered with Kosambi mapping function of JoinMap v. 4.0 software using a LOD score ≥ 3 after preliminary analysis using a LOD score ranging from 2 to 10. MapChart2.2^6^ was used to draw the genetic linkage map. For the redundant loci that showed co-segregation in the 163 SK-RILs, only one unique informative marker is shown. The short chromosome arms are on the top.

### QTL Analysis

The SK-RIL population derived genetic linkage map was used to screen QTL in this study. The average trait phenotypic values of two replicates under each environment (E1–E8) were used for individual environment QTL analysis. The inclusive composite interval mapping (ICIM) performed with IciMapping 4.1 was conducted to detect putative additive QTL. Moreover, Multi-environment Traits (MET) analysis was employed for combined QTL analysis across environments to verify the QTL identified in the individual environment and evaluate the QTL × environment interactions. The conditional phenotypic values evaluated by QGAStation 2.0 were used for conditional QTL mapping analysis. The missing phenotypic data were deleted using the ‘Deletion’ command. The walking speed for all QTL was 1.0 cM, and the *P*-value inclusion threshold was 0.001. The threshold LOD scores were calculated using 1000 permutations with a type I error of 0.05 (Doerge and Churchill, 1996; Li et al., 2007). A suggestive QTL with an average LOD value > 2.5 in a data set was shown. A QTL with an average LOD value > 3.0 and average phenotypic variance contribution > 10% was defined as a major QTL, and one showing significance in at least four environments sets was considered a stable QTL (Lander and Kruglyak, 1995; Cui et al., 2016).

## Results

### High-Density Genetic Linkage Map Construction

Genotyping of the SX828/KN2007 RIL population with the iSelect 90K SNP array resulted in 10,638 (13.04%) polymorphic markers. In addition to the SNP markers, 225 SSR markers were also used for the linkage analysis. After removing ambiguous and unlinked markers, a genetic linkage map with 6312 markers (including 6130 SNP markers and 182 SSR markers) was constructed, which was within 2672 unique loci, spanning 3049.4 cM in length with an average marker density of 1.1 cM/locus, covering 21 wheat chromosomes (**Table 1**, **Supplementary Table S2**, and **Supplementary Figure S1**). Of the 6130 SNP markers, 6118 (99.8%) were best hits to 4100 Chinese Spring (CS) sequence contigs, with 1.5 polymorphic markers per contig. In total, 79.8% of the contigs had coincident physical and genetic positions, while 16.6% of the contigs were mapped to the homoeologous chromosomes such as 1A in physical position but 1B in the SK-RIL genetic map, and the rest of 3.6% were disordered (**Supplementary Table S3**). All together, of the 6312 high-quality polymorphic markers, 2565 (40.6%) were localized to the A genome spanning 1059.1 cM with an average marker density of 0.41 cM/locus, 2919 (46.2%) were mapped to the B genome covering 1140.7 cM with an average marker density of 0.39 cM/locus, and 828 (13.2%) were mapped to the D genome spanning 849.6 cM with an average marker density of 1.03 cM/locus. 86.8% of the markers in total mapped to the A and B genomes, revealed higher polymorphisms of the A and B genomes than that of the D genome. The 6312 markers distributed unevenly on the 21 chromosomes, ranging from 18 on chromosome 4D to 790 on chromosome 5B. Chromosomes 1B, 2D, and 5D were integrated by two linkage groups, respectively. Ten gaps (>20 cM) were found on chromosomes 1D, 2B (2), 3B, 4B, 5D, 6D, 7B and 7D (2) (**Supplementary Table S2** and **Supplementary Figure S1**).

**Table 1:** General information for the high-density genetic linkage map.

Chromosome	Total marker	SNP	SSR	Bin	Map length	Marker density
	numbers	markers	markers		(cM)	(cM/Marker)
1A	432	427	5	145	126.2	0.9
1B^‡^	220	215	5	107	105.6	1.0
1D	105	95	10	70	146.7	2.1
2A	493	487	6	185	157.8	0.9
2B	698	678	20	271	196.9	0.7
2D^‡^	346	331	15	127	255.1	2.0
3A	216	215	1	129	121.5	0.9
3B	399	384	15	195	188.1	1.0
3D	86	85	1	22	76.3	3.5
4A	138	132	6	85	145.1	1.7
4B	103	99	4	58	131.0	2.3
4D	18	16	2	16	48.1	3.0
5A	329	318	11	152	108.8	0.7
5B	790	767	23	263	150.5	0.6
5D^‡^	128	126	2	47	97.4	2.1
6A	421	411	10	184	178.3	1.0
6B	473	461	12	237	175.8	0.7
6D	99	99	0	32	133.2	4.2
7A	536	515	21	229	221.4	1.0
7B	236	223	13	98	192.8	2.0
7D	46	46	0	20	92.8	4.6
Genome A	2565	2505	60	1109	1059.1	1.0
Genome B	2919	2827	92	1229	1140.7	0.9
Genome D	828	798	30	334	849.6	2.5
Total	6312	6130	182	2672	3049.5	1.1


*^‡^Chromosomes with two separated linkage groups.*

### Phenotypic Variation and Correlation Analysis

The phenotypic values of the SK-RILs and parents are shown in **Table 2**. In all eight tested environments, the parent SX828 exhibited higher TKW and longer kernel than that of the other parent KN2007, while KN2007 had wider kernel than SX828. All of the investigated traits manifested continuous segregation in the RIL population, and the most absolute values of skewness and kurtosis for all investigated traits were less than 1.0, indicating normal distribution and involvement of multiple genes for these traits. Strong transgressive segregation exceeding the limits of both parents was observed, suggesting that alleles with positive effects were distributed between the two parents. High value of broad sense heritability ($h_{B}^{2}$) for all the investigated traits suggested that genetic factor played an important role in the formation of these traits (**Table 2**).

**Table 2:** Phenotypic values for yield traits in the two parents and SK-RILs.

Trait ^a^	En^b^	*N*	Parents		SK-RILs						
				
		plots	SX828	KN2007	Min. (cm)	Max. (cm)	Mean ± SD (cm) ^c^	Skewness	Kurtosis	K-S *P* Value ^d^	$h_{B}^{2}$
KL	E1	HN	6.63	5.63	5.38	6.70	6.08 ± 0.30	-0.08	-0.76	0.01	0.82
	E2	HN	6.80	5.58	5.49	7.00	6.26 ± 0.33	0.08	-0.70	0.03	0.85
	E3	LN	6.93	5.59	5.43	6.93	6.23 ± 0.32	-0.01	-0.58	0.07	0.93
	E4	HN	6.64	5.46	5.45	7.02	6.23 ± 0.32	0.16	-0.33	0.10	0.39
	E5	LN	6.72	5.67	5.54	7.05	6.29 ± 0.32	0.06	-0.46	0.08	0.91
	E6	HN	6.62	5.39	5.29	6.62	5.88 ± 0.32	0.13	-0.61	0.00	0.89
	E7	HN	6.89	5.75	5.47	6.95	6.27 ± 0.31	0.14	-0.59	0.07	0.67
	E8	LN	6.82	5.82	5.55	6.97	6.28 ± 0.31	0.05	-0.28	0.13	0.67
KW	E1	HN	3.58	3.72	2.97	3.95	3.41 ± 0.15	0.04	0.72	0.96	0.52
	E2	HN	3.72	3.80	3.31	4.18	3.76 ± 0.13	-0.20	1.12	0.92	0.42
	E3	LN	3.77	3.81	3.36	4.36	3.75 ± 0.12	0.52	4.18	0.06	0.79
	E4	HN	3.34	3.34	3.04	3.85	3.44 ± 0.13	-0.11	0.23	0.99	0.42
	E5	LN	3.54	3.59	3.24	3.91	3.54 ± 0.11	0.00	0.46	0.82	0.73
	E6	HN	3.33	3.48	2.79	3.64	3.25 ± 0.16	0.00	-0.22	0.75	0.57
	E7	HN	3.52	3.72	3.22	3.90	3.50 ± 0.12	0.13	-0.04	0.75	0.83
	E8	LN	3.41	3.75	3.13	3.91	3.45 ± 0.13	0.20	0.53	0.97	0.89
KDR	E1	HN	1.87	1.52	1.50	2.06	1.79 ± 0.10	-0.05	0.11	0.85	0.92
	E2	HN	1.84	1.48	1.39	1.97	1.68 ± 0.10	0.21	0.17	0.78	0.82
	E3	LN	1.85	1.47	1.31	1.91	1.67 ± 0.10	-0.04	0.63	0.78	0.96
	E4	HN	2.01	1.64	1.49	2.10	1.83 ± 0.10	0.15	0.13	0.78	0.79
	E5	LN	1.91	1.58	1.45	2.01	1.78 ± 0.10	0.15	0.06	0.23	0.92
	E6	HN	2.01	1.56	1.50	2.11	1.82 ± 0.10	0.09	0.41	0.76	0.86
	E7	HN	1.97	1.56	1.47	2.07	1.80 ± 0.11	0.06	0.05	0.48	0.34
	E8	LN	2.01	1.55	1.46	2.14	1.84 ± 0.12	0.04	0.12	0.67	0.76
TKW	E1	HN	50.55	49.29	27.44	56.54	44.38 ± 4.94	-0.24	0.23	0.89	0.75
	E2	HN	55.22	48.95	40.37	63.94	52.33 ± 4.37	0.06	0.02	0.79	0.53
	E3	LN	59.67	51.80	43.50	63.70	53.73 ± 4.16	0.01	-0.38	0.31	0.78
	E4	HN	46.39	39.45	32.61	54.52	42.21 ± 4.00	0.29	0.15	0.53	0.69
	E5	LN	46.38	41.11	34.19	51.99	43.34 ± 3.24	-0.03	-0.23	0.83	0.71
	E6	HN	48.05	43.96	32.53	53.76	42.41 ± 3.61	0.01	0.39	0.88	0.59
	E7	HN	46.10	41.72	34.19	52.64	41.95 ± 3.52	0.33	-0.01	0.23	0.82
	E8	LN	43.28	43.87	32.27	53.25	40.44 ± 3.73	0.40	0.25	0.15	0.85
SNPP	E1	HN	10.00	11.33	8.00	20.67	18.07 ± 3.71	0.68	1.31	0.03	0.44
	E2	HN	9.70	10.30	7.10	15.60	11.85 ± 1.77	0.13	-0.43	0.04	0.62
	E3	LN	4.40	5.30	3.80	8.40	5.36 ± 0.79	0.93	1.34	0.00	0.68
	E4	HN	14.90	15.10	11.10	23.00	15.79 ± 2.25	0.55	0.48	0.01	0.49
	E5	LN	4.80	6.70	4.50	9.30	6.65 ± 0.95	-0.07	-0.15	0.24	0.57
	E6	HN	9.90	11.30	9.20	15.60	12.08 ± 1.30	0.10	-0.21	0.26	0.38
	E7	HN	9.00	10.60	7.60	13.30	10.12 ± 1.07	0.10	-0.21	0.52	0.49
	E8	LN	12.00	11.90	8.70	16.70	11.57 ± 1.49	0.75	0.87	0.00	0.50
KNPS	E1	HN	53.00	56.00	27.00	78.33	59.02 ± 8.28	-0.44	0.76	0.65	0.67
	E2	HN	60.10	54.40	41.70	82.50	60.40 ± 6.86	0.19	-0.14	0.82	0.77
	E3	LN	49.80	58.70	42.80	77.60	59.06 ± 6.39	0.32	0.17	0.46	0.72
	E4	HN	63.70	52.60	39.30	74.50	55.77 ± 6.36	0.17	-0.16	0.75	0.66
	E5	LN	54.40	58.20	38.70	76.10	57.22 ± 6.35	0.38	0.15	0.01	0.76
	E6	HN	61.80	63.40	44.30	79.10	60.00 ± 6.15	0.15	0.21	0.74	0.82
	E7	HN	76.00	78.00	53.20	99.60	71.00 ± 7.39	0.35	0.70	0.79	0.82
	E8	LN	71.40	70.00	59.30	101.90	74.66 ± 7.52	0.62	0.67	0.03	0.80


*^*a*^KL, kernel length; KW, kernel width; KDR, kernel diameter ratio; TKW, thousand kernel weight; SNPP, spike number per plant; KNPS, kernel number per spike. ^*b*^E1, E2, E3, E4, E5, E6, E7, and E8 indicate the trails were conducted in: 2014–2015, Shijiazhuang, high nitrogen (HN); 2015–2016, Shijiazhuang, HN; 2015–2016, Shijiazhuang, low nitrogen (LN); 2016–2017, Shijiazhuang, HN; 2016–2017, Shijiazhuang, LN; 2016–2017, Anyang, HN; 2016–2017, Beijing, HN and 2016–2017, Beijing, LN, respectively. ^*c*^Five representative plants in the center of the second row were used to estimate the mean value. ^*d*^*P*-value was acquired through Kolmogorov–Smirnov test to testify the assumption of normality.*

Pearson’s coefficients of correlation were calculated for all the traits based on the average data of the eight environments (**Supplementary Table S4**). TKW was significantly and positively correlated with KL and KW. The correlation coefficient of KW-TKW (*r* = 0.696, *P* < 0.01) was higher than that of KL-TKW (*r* = 0.592, *P* < 0.01), suggesting that KW should be the main contributor to the increased grain weight. Furthermore, SNPP was significantly and positively correlated to KDR but negatively correlated to KW, while KNPS was significantly negatively correlated to KL, TKW and SNPP.

### Putative Additive QTL for Kernel Traits

A total of 78 putative additive QTL associated with KL, KW, KDR, and TKW were detected in the eight individual environments (**Table 3** and **Supplementary Table S5**). It was found that these QTL were distributed across all wheat chromosomes except for 3D, 4D, and 7D. Of these, 32 QTL were mapped to the A genome, 30 to the B genome and 16 to the D genome. These QTL individually explained 3.95–16.57% of the phenotypic variance with LOD value ranging from 2.60 to 10.59. Thirty-three QTL (42.31%) were reproducibly detected in at least two environments. Sixteen QTL (20.51%) individually accounted for more than 10% of the phenotypic variance with an average LOD value > 3.0 (defined as major QTL); three of these (*qKW-2D.1, qKDR-2D.1* and *qTKW-5B.2*) also showed stability across more than four environments and were characterized as major and stable QTL. All of the six stable QTL identified in the individual environment were verified in combined analysis across eight environments (**Table 3**).

**Table 3:** Partial stable QTL for TKW and kernel size.

QT L	Interva	En ^a^	LOD ^b^	PVE (%) ^c^	Add ^d^	LOD	LOD	PVE	PVE	Add
	l (cM)					(A) ^e^	(A by E) ^f^	(A) ^g^	(A by E) ^h^	(A) ^i^
*qKL-2D*	123.95–132.38	E1/E3/E4/E5/	3.62/2.27/4.43/9.92/	4.58/4.27/6.88/10.14/	0.06/0.07/0.08/0.10/	9.84	8.39	1.61	1.27	0.04
		E7/E8	3.65/6.48	5.24/9.85	0.07/0.10					
*qKL-6B.2*	86.80–91.22	E1/E2/E3/	6.76/7.05/6.60/	12.25/10.11/11.12/	0.11/0.10/0.11/	25.34	8.99	4.21	1.86	0.07
		E6/E7	5.84/3.38	8.77/4.89	0.09/0.07					
*qKW-2D.1*	121.79–128.59	E1/E2/E3/E5/	3.59/5.20/6.57/10.07/	7.55/9.34/13.54/12.52/	-0.04/-0.04/-0.04/-0.04/	11.39	13.87	2.30	2.19	-0.02
****		E6/E7/E8	5.08/10.25/6.03	7.01/18.82/16.29	-0.04/-0.05/-0.05					
*qKDR-2D.1*	124.89–127.13	E1/E2/E3/E4/	7.83/12.47/12.01/11.70/	13.78/16.39/18.22/15.54/	0.04/0.04/0.04/0.04/	67.04	1.69	14.84	0.11	0.04
****		E5/E6/E7/E8	12.81/7.88/8.11/8.93	16.07/17.27/17.15/18.10	0.04/0.04/0.05/0.05					
*qTKW-5A*	16.92–17.29	E1/E2/E4/E6	2.57/5.34/2.57/2.54	5.30/8.07/4.80/4.60	1.14/1.25/0.88/0.78	16.88	9.37	3.43	1.92	0.74
*qTKW-5B.2*	50.97–55.01	E2/E4/E5/	9.34/5.68/15.05/	14.72/11.12/24.41/	1.68/1.34/1.60/	11.19	20.24	2.29	3.03	0.60
		E6/E7	5.94/3.01	11.13/10.04	1.21/1.15					


*^*a*^E1, E2, E3, E4, E5, E6, E7, and E8 indicate trial 1 (2014–2015, Shijiazhuang) HN, trial 2 (2015–2016, Shijiazhuang) HN, trial 2 LN, trial 3 (2016–2017, Shijiazhuang) HN, trial 3 LN, trial 4 (2016–2017, Anyang) HN, trial 5 (2016–2017, Beijing) HN, and trial 5 LN, respectively. ^*b,c,d*^Values of the corresponding additive QTL in individual environment. A positive sign indicates alleles from SX828 increased the corresponding trait value, and a negative sign indicates alleles from KN2007 increased the corresponding trait value. PVE, phenotypic variance explanation; Add, additive effect. ^*e,g,i*^ Values of the corresponding additive QTL in combined QTL analysis across environments. ^*f,h*^Values for interaction effect of A (additive) by E (environment) of the corresponding additive QTL in combined QTL analysis across environments.*

Eighteen QTL for KL were identified on chromosomes 1A, 1D, 2A, 2B (3), 2D, 3A (2), 3B, 4A, 5B (3), 6B (2), 7A and 7B, respectively, with individual QTL explaining 4.14–16.56% of the phenotypic variance. Of these QTL, 14 carried the favorable alleles increasing KL from SX828. Two stable QTL, *qKL-2D* and *qKL-6B.2*, individually showed significant effects in six and five environments and exhibited 4.27–10.14% and 4.89–12.25% of the phenotypic variance, respectively. Favorable alleles of the two QTL were contributed by SX828.

Twenty QTL for KW were detected on chromosomes 1A (2), 1D, 2D (2), 3A, 3B (2), 4A, 4B (3), 5A, 5B, 5D, 6A (2), 6D and 7B (2), respectively, with individual QTL explaining 4.63–15.51% of the phenotypic variance. Alleles increasing KW at 12 loci were derived from the parent KN2007. For *qKW-2D.1*, a major and stable QTL exhibiting 7.01–18.82% of the phenotypic variance with LOD value of 3.59–10.25, the favorable allele was derived from KN2007. For *qKW-4B.1* and *qKW-5B* which were repeatedly identified in at least three different environments, the alleles increasing KW were from SX828.

Nineteen QTL for KDR were identified on chromosomes 1D (3), 2B (2), 2D (2), 3A, 4A (2), 5A (3), 5B (2), 6A (3) and 6B respectively, with individual QTL explaining 4.17–16.57% of the phenotypic variance. Of these, 10 QTL were repeatedly identified in at least two different environments. Except for *qKDR-2B.1*, all of the alleles that increased KDR in these loci came from SX828. *qKDR-2D.1*, co-localized with *qKL-2D* and *qKW-2D.1* and flanked by markers *Xcfd168* and *BobWhite_c7149_371*, was identified in all eight environments and explained 13.78–18.22% of the variation in KDR with LOD value of 7.83–12.81.

Twenty-one QTL for TKW were detected on chromosomes 1A (2), 1B, 2A (2), 2B, 2D (3), 3A (3), 3B, 4B, 5A, 5B (3), 6A, 6D and 7A, respectively, with individual QTL explaining 3.95-15.34% of the phenotypic variance. *qTKW-5A* and *qTKW-5B.2* were significant in at least four environments, individually exhibiting 4.60–8.07% and 10.04–24.41% of the phenotypic variance, respectively. *qTKW-5B.2*, a major and stable QTL, was co-localized with *qKL-5B.1* and the favorable allele from SX828 simultaneously increased TKW and KL.

### Clusters With Co-located QTL for Kernel-Related Traits

Overview of the identified QTL for kernel traits in this study, 7 QTL clusters (C1–C7) comprising 17 QTL were scattered on chromosomes 1A (two clusters), 2D, 3A, 4B and 5B (two clusters) (**Table 4** and **Supplementary Figure S1**). Among the clusters, QTL for TKW were always identified together with QTL for kernel size and shape, except for C3 where only QTL for KL, KW, and KDR were co-located.

**Table 4:** Characterization of QTL clusters for kernel traits in this study.

Cluster	Chromosome	Interval (cM)	QTL included	Traits (additive effect,
				number of environments) ^a^
C1	1A	48.93–57.29	*qKW-1A.1*; *qTKW-1A.1*	KW(-, 1), TKW(-, 2)
C2	1A	66.97–73.21	*qKW-1A.2*; *qTKW-1A.2*	KW(-, 1), TKW(-, 2)
C3	2D	121.79–132.38	*qKL-2D*; ***qKW-2D.*1**; ***qKDR-2D.1***	KL(+, 6), **KW(-, 7)**, **KDR(+, 8)**
C4	3A	0–8.60	*qKW-3A*; ***qTKW-3A.1***	KW(-, 1), **TKW(-, 1)**
C5	4B	21.77–30.39	*qKW-4B.1*; *qTKW-4B*	KW(+, 3), TKW(+, 1)
C6	5B	35.53–45.36	*qKW-5B; qKDR-5B.1*; *qTKW-5B.1*	KW(+, 3), KDR(+, 2), TKW(+, 2)
C7	5B	49.82–59.16	***qKL-5B.1***; *qKDR-5B.*2; ***qTKW-5B.2***	**KL(+, 3)**, KDR(+, 2), **TKW(+, 5)**


*^*a*^The trait name in bold type indicates that major QTL were detected for the corresponding trait; the trait name in underlined type indicates that stable QTL were detected for the corresponding trait.*

C3 was positioned on chromosome 2D with co-existed *qKL-2D, qKW-2D.1*, and *qKDR-2D.1*. The stable QTL *qKL-2D* was detected in six environments, with SX828-derived alleles increasing KL and KDR. The major and stable QTL *qKW-2D.1* was reproducibly identified in seven environments, with the favorable alleles from KN2007 increasing KW. The stable QTL *qKDR-2D.1* showed significance in all eight environments. Interestingly, the positive alleles of co-located QTL for KL and KW in C3 were derived from opposite parent, which may result in no significant QTL for TKW identified.

In the other six clusters, the positive alleles of the co-located QTL for KL/KW/KDR and TKW were consistently contributed by single parent. For C1 and C2 on chromosome 1A, *qTKW-1A.1* and *qTKW-1A.2* were reproducibly identified in different environments, while QTL for KW in the two clusters were significant in only one environment. Favorable alleles of those QTL were all from KN2007. For C4 on chromosome 3A, a major QTL *qTKW-3A.1* was clustered with a QTL *qKW-3A*, with the KN2007-derived alleles simultaneously increasing KW and TKW. For C5 on chromosome 4B, *qKW-4B.1* was co-localized with *qTKW-4B*, with the favorable alleles from SX828 increasing KW and TKW. For C6 on chromosome 5B, *qKW-5B* showed significance in three environments and clustered with *qKDR-5B.1* and *qTKW-5B.1*, with favorable alleles from SX828. For C7 on chromosome 5B, *qKL-5B.1, qKDR-5B.2* and *qTKW-5B.2* were clustered. The major and stable QTL *qTKW-5B.2* was expressed in five environments, with alleles from SX828 simultaneously increasing KL, KDR, and TKW.

### Conditional QTL for TKW With Respect to TKWC

To dissect the genetic effects of TKWC on the expression of QTL for the TKW that were detected in the aforementioned 7 clusters, conditional QTL mapping analysis for the TKW was conducted with respect to TKWC.

Unconditional and conditional QTL mapping results revealed that KL, KW, and KDR played different roles in the expression of QTL for TKW detected in C1-C7 (**Table 5**). *qTKW-1A.1, qTKW-3A.1*, and *qTKW-4B.1* were found to be related to the variation of all three elements. *qTKW-1A.2* and *qTKW-5B.1* were entirely contributed by KW and KDR and partially contributed by KL. *qTKW-5B.2* was found entirely due to the variation of KL and in part to the variation of KW and KDR. *qTKW-2D.4* was a new QTL for TKW only detected in conditional QTL analysis (**Table 5**). When TKW conditional on the KL (TKW| KL), i.e., removing off the effect of KL on TKW, the additive effect of *qTKW-2D.4* was derived from KN2007. Likewise, using the conditional data of TKW| KW, the additive effect of *qTKW-2D.4* was derived from SX828.

**Table 5:** Conditional QTL for TKW with respect to kernel size.

Cluster ^a^	QTL ^b^	Interval markers ^c^	Unconditional QTL Additive [En/PVE(%)] ^d^	Conditional QTL Additive [En/PVE(%)]		
				
			TKW	TKW| KL	TKW| KW	TKW| KDR
C1	*qTKW-1A.1*	*Wp_CAP12_c2438_1180601*—*Ex_c2389_1834*				-1.05 (E4/6.78)
			-0.85 (E6/5.15)			
			-0.71 (E7/3.96)			
C2	*qTKW-1A.2*	*Xgwm164—Xgwm135*	-1.02 (E2/5.37)	-1.04 (E2/9.33) =		
			-1.24 (E4/9.54)	-0.92 (E4/7.13) -		
						
C3	*qTKW-2D.4*	*Xwmc181.2—BS00062567_51*		-1.36 (E1/12.42)	0.52 (E1/4.64)	
				-1.25 (E2/13.26)		
				-1.23 (E4/12.57)	0.74 (E4/8.01)	
				-0.78 (E5/9.28)	0.76 (E5/12.17)	
				-0.68 (E6/5.39)		
				-1.25 (E7/16.62)		
				-0.90 (E8/7.32)	0.49 (E8/4.29)	
C4	*qTKW-3A.1*	*Exb_c32653_553—RFL_cg1896_1236*			-0.61 (E5/5.99)	
			-1.38 (E7/13.74)	-0.98 (E7/8.36) -		
C5	*qTKW-4B.1*	*BS00068104_51—Kukri_c52413_282*		0.75 (E3/5.70)		
			0.64 (E5/3.95)			0.95 (E5/8.54)+
C6	*qTKW-5B.1*	*CAP7_c5481_96—Xwmc386*	0.99 (E7/7.92)	0.93 (E7/9.17) =		
			1.24 (E8/10.27)			
C7	*qTKW-5B.2*	*BS00050775_51—Exb_c37146_747*	1.68 (E2/14.72)		1.26 (E2/16.76) -	1.44 (E2/11.05) -
					1.23 (E3/14.41)	
			1.34 (E4/11.12)			1.50 (E4/14.01)+
			1.60 (E5/24.41)		0.81 (E5/13.58) -	1.35 (E5/17.75) -
			1.21 (E6/11.13)		1.07 (E6/13.02) -	
			1.15 (E7/10.04)			
						1.12 (E8/9.14)


*^*a*^QTL for kernel-related traits located in the 7 clusters in **Table 4**. A putative major QTL is marked by bold typeface which is characterized by a mean LOD value > 3.0 and a mean PVE > 10%; a putative stable QTL is underlined when this locus can be detected in at least four of the eight environments. ^*b*^The clusters containing QTL affecting kernel-related traits are shown in **Table 4**. ^*c*^Flanking markers of the QTL. ^*d*^Numerals before parentheses are estimated of the additive effects of the QTL. Positive values indicate that SX828 alleles increase the TKW. Negative values indicate that KN2007 alleles increase TKW. E and numerals in parentheses indicate the environment in which the QTL was detected and the percentage of phenotypic variance explained by the additive effects of the mapped QTL, respectively. A minus sign, “-”, or a plus sign, “ + ”, following the parentheses denotes the additive effect of a conditional QTL, in absolute values, that reduces or increases more than 10 % compared to the corresponding unconditional QTL, respectively. An equal sign, “ = ”, is placed after the parentheses to denote a conditional QTL with an equal additive effect to that of the unconditional.*

## Discussion

### High-Density Genetic Linkage Map Construction

SNP markers enable construction of high-density genetic linkage maps and identification of QTL for complex agronomic traits in crop plants. In the present study, a genetic linkage map was constructed comprising 6130 SNP markers and 182 SSR markers (**Table 1**, **Supplementary Table S2**, and **Supplementary Figure S1**). It is noteworthy that 951 (15.5%) SNP markers of iSelect 90K array were newly mapped (**Supplementary Table S6**). Order of the SNP in the present genetic map was in good agreement with that in the recently released wheat genome assembly (**Supplementary Table S3**). Notably, the high number (10,638) of polymorphic SNP markers between SX828 and KN2007 is comparable to Gao et al. (2015) and Zhai et al. (2016), who detected 7514 and 11,646 polymorphic SNPs between the two parental lines by iSelect 90K array, respectively. The relatively high SNP polymorphism in our mapping population confirmed the genetic divergence between the two parental lines. The genetic length of this map is 3049.4 cM, similar to the reported maps in hexaploid wheat (Somers et al., 2004; Wu et al., 2015; Zhai et al., 2016). In consequence, the current genetic map based on iSelect 90K array would be good enough for QTL mapping.

The number of markers on each genome was uneven. Markers for the A (40.6%) and B (46.2%) genomes were more abundant than those for the D genome, consistent with previous studies (Somers et al., 2004; Chao et al., 2009; Wang et al., 2014), and this is attributed to the low level of polymorphism in the D genome of hexaploid wheat. D genome is a recent evolutionary addition to the hexaploid wheat genome and there has been limited gene flow *Aegilops tauschii* Coss. and *T. aestivum* (Dubcovsky and Dvorak, 2007), possibly explaining the low polymorphism rate.

### Response of QTL for Kernel Traits to Divergent Nitrogen Supply

Nitrogen (N) is an essential mineral nutrient required by crop plants. Sufficient N supply usually resulted in increased productive tillers, kernel number per spike and grain yield, which are frequently accompanied with smaller kernel size and lower kernel weight (Sinclair and Jamieson, 2006; Makino, 2011). In our study, SNPP were negatively correlated with KW, and KNPS exhibited significant negative correlation with KL and KW (**Supplementary Table S4**). Most values of kernel size and kernel weight of SK-RILs under LN supplies were higher than those under HN supplies, which is in agreement with the negative correlation between grain size and nitrogen supplies (Habash et al., 2007; Makino, 2011).

Genomic regions detected under a specific nitrogen treatment are more probably involved in wheat adaptation to the corresponding environment (Laperche et al., 2007; Fan et al., 2015). It can be seen that five stable QTL (*qKL-2D, qKL-6B.2, qKW-2D.1, qKDR-2D.1* and *qTKW-5B.2*) for kernel traits were expressed in both HN and LN environments (**Table 3** and **Supplementary Tables S5, S7**), indicating that the expression of these QTL were less sensitive to nitrogen supply. 36 QTL including one stable QTL *qTKW-5A* were only detected under HN condition means that the expression of these QTL were induced by HN supply (**Supplementary Tables S5, S7**). Seventeen QTL were expressed only under LN condition indicates that these QTL were adapting to nitrogen constraint (**Supplementary Table S5**). Notably, the *qTKW-5A* is valuable in obtaining higher grain weight under the sufficient N supply management, and the allele from SX828 may increase the nitrogen uptake and use efficiency (**Supplementary Table S7**).

### Pleiotropic Cluster on Chromosomes 2D

**FIGURE 1:**
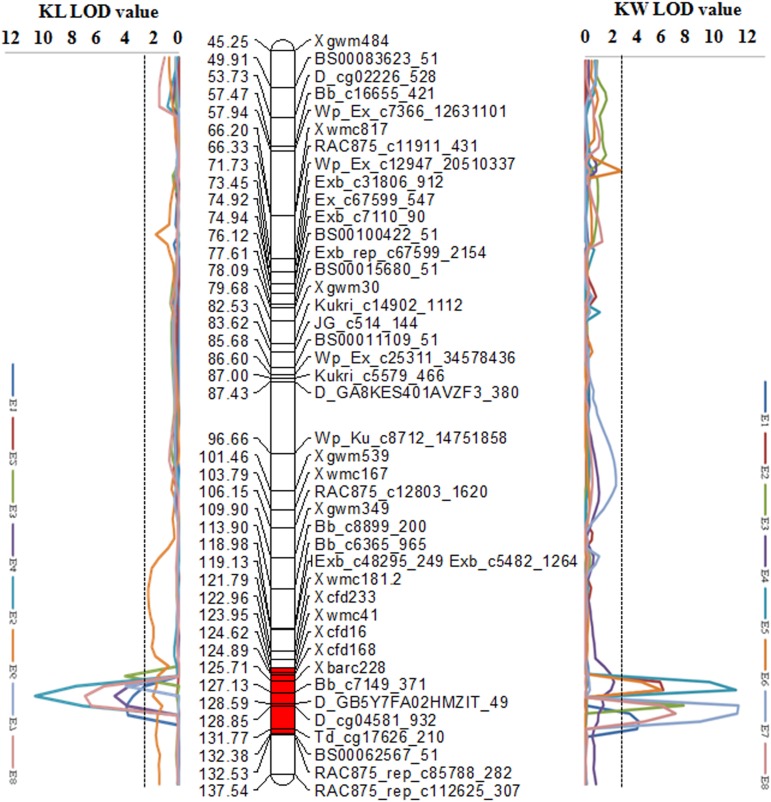
The LOD value of QTL on chromosome 2D for kernel length and kernel width in eight environments. The LOD value of black dashed line is 2.5. The map on the left is the LOD value of QTL for kernel length in eight environments; the map on the middle is the high-resolution genetic linkage map of chromosome 2D; the map on the right is the LOD value of QTL for kernel width in eight environments.

Associative traits are prone to share regions with significant QTL. Several genetic loci simultaneously controlling KL and KW were identified on chromosome 2D in cluster 3 (**Table 4** and **Figure 1**). These loci have also been highlighted in other studies. Campbell et al. (1999) firstly reported a RFLP marker linked to KL on 2D, later Breseghello and Sorrells (2006) detected a KL-associated 2D marker *Xgwm539*, which is close to *qKL-2D.1* in our study. However, Ramya et al. (2010) reported that *Xgwm539* was linked to *QTkw.ncl-2D.2* and *QKw.ncl-2D.2*. In our study, QTL in cluster 3 were only for KL and KW with no significant effect on TKW (**Table 4**, **Supplementary Table S5**, and **Supplementary Figure S1**). Nevertheless, when the influence of KL or KW on TKW was excluded, a stable QTL (*qTKW-2D.4*) for TKW was detected (**Table 5**), suggesting a tension or tradeoff between the two kernel dimensions. This finding is not at all accidental. In previous QTL analyses, increasing effects of QTL on respective traits have frequently been supplied by different parents in an opposite manner (Liu et al., 2014; Cheng et al., 2017). A comparison of the QTL on chromosome 2D detected in the present study to those identified in previous studies showed that these important QTL were located at an approximately equivalent or adjacent chromosomal region (**Supplementary Figure S2**). Moreover, *Rht8*, one of gibberellin-responsive (GAR) dwarfing genes tightly linked with *WMC503* and *XGWM261* (Korzun et al., 1998; Du et al., 2018), was mapped on chromosome 2DS (**Supplementary Figure S1**). There was a large interval between cluster 3 and *WMC503*/*XGWM261*. Thus, we suppose there is no linkage between the C3 (**Table 4**) and *Rht8* in our study. This region of chromosome 2D should be good donor for improving kernel size, to which should be paid more attention in wheat breeding programs.

We previously mapped *TaGS2-D1* to the same region on 2DL in the KJ-RIL map (Cui et al., 2014) and found that *TaGS2-D1* from KN9204 had positive effects on KW, KDR and TKW (Cui et al., 2016). Therefore, we used *IN10*, one functional marker of *GS2*, to genotype SX828 and KN2007 and detected two banding patterns. KN2007 and KN9204 displayed identical banding pattern while SX828 and J411 were the same (**Supplementary Figure S3**), which implied that KN2007 might share the same chromosome interval of 2D as KN9204, thus the C3 on chromosome 2D of this study (**Table 4**) might harbor the similar QTL related with kernel size on chromosome 2D of the KJ-RIL population (Cui et al., 2016). As KN2007 was derived from KN9204 and the favorable allele *qKW-2D* was donated by KN2007, the accomplishing of whole genome sequence assembly of KN9204 (not published) will benefit fine-mapping of this major QTL cluster.

### QTL for TKW on Chromosome 5B and the Prediction of Candidate Genes

Another notable cluster C7 on chromosomes 5B consisted of a range of QTL affecting KL, KDR and TKW (**Table 4**). The results of conditional QTL mapping in the present study also indicated that *qTKW-5B.2* was entirely explained by KL and partially contributed by KW (**Table 5**). In addition, *qTKW-5B.2* was detected when conditional analysis was conducted on SNPP and KNPS (**Supplementary Table S8**). These data suggested that the expression of *qTKW-5B.2* was primarily dependent on variation of KL. A number of QTL for TKW and kernel size on chromosome 5B have been reported in previous studies (Groos et al., 2003; Breseghello and Sorrells, 2006). Among them, *QTkw.ncl-5B.2* and *QTw-5B.1* associated with *Xgwm213* were located on the 5BL (Ramya et al., 2010); *QTkw5B.1-12* and *QKw5B.1-12* were located on 5B (Li et al., 2015). Sarma et al. (2000) reported two possible loci affecting flowering time on the long arm of the 5B chromosome (*Vrn-B1* and *Eps*). Tóth et al. (2003) found that *Vrn-B1* was closely linked to the SSR marker *Xgwm604* and *Eps-5BL2* was linked to the SSR locus *Xwmc73*. However, *Xgwm604* and *Xwmc73* were no polymorphism in our SK-RILs (**Supplementary Figure S4**). Thus, we supposed this loci should not be association with flowering time in our study. After comparing the position of the marker intervals, *qTKW-5B.2* identified in the SK-RIL population was considered preliminarily as a new QTL for its large distance from other reported QTL. Therefore, further fine mapping of the putative pleiotropic QTL in this interval is of great value.

**FIGURE 2:**
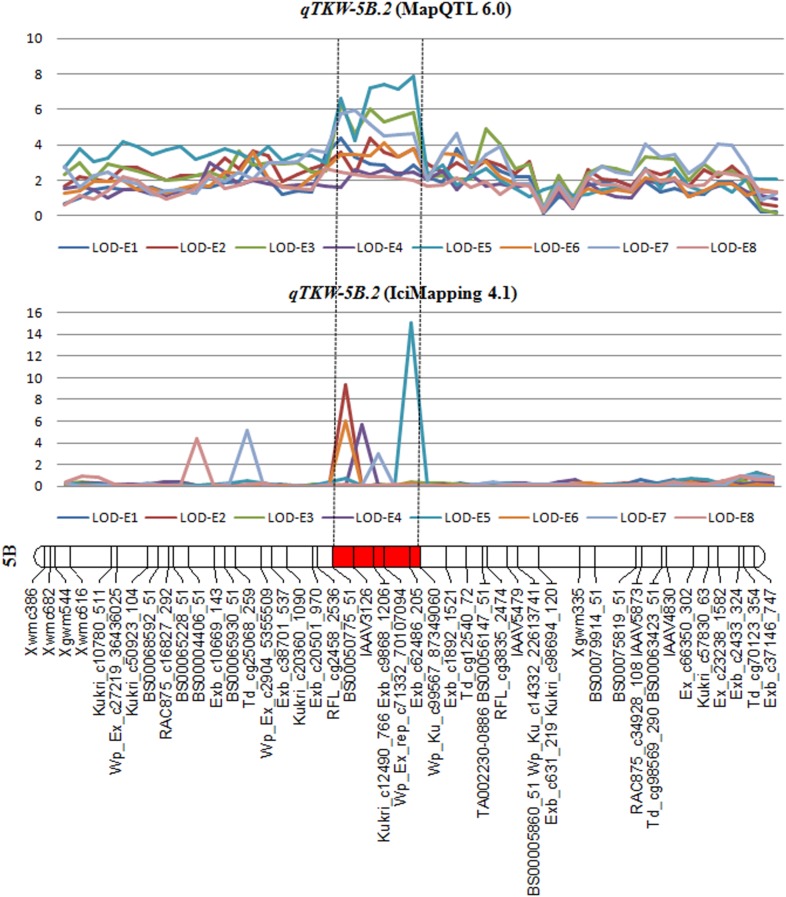
Overlapping confidence interval of QTL for TKW based on MapQTL 6.0, IciMapping 4.1 and QTLNetwork 2.0. The segment in red is the overlapping confidence interval of *qTKW-5B.*

*qTKW-5B.2* was repeatedly identified using various softwares based on different mathematical models (**Table 6** and **Figure 2**). The peak position of this QTL was found in the *BS00050775_51* – *IAAV3126* overlapping confidence interval of 0.7 cM. Based on the genome sequence assembly of *T. aestivum* cv. Chinese Spring^7^, the overlapping confidence intervals of *BS00050775_51* - *IAAV3126* spanned 45.41 Mb (5B: 236261222 – 5B: 281675808) in physical position (**Supplementary Table S3**) with 357 predicted protein coding genes in wheat (**Supplementary Figure S5**). This information is very valuable for future high-resolution mapping and map-based cloning of *qTKW-5B.2*.

**Table 6:** *qTKW-5B.2* as detected by MapQTL 6.0, IciMapping 4.1 and QTLNetwork 2.0.

Software	LOD value	Position (cM)	Additive effect	PVE %
MapQTL 6.0	1.49–7.90	50.05–55.80	0.75–1.76	4.10–19.80
IciMapping 4.1	5.68–15.05	50.97–55.01	1.15–1.68	10.04–24.41
QTLNetwork 2.0	*P-*Value: 0.000000	51.0	1.3	4.83


Using *BS00050775_51* as a probe, the 163 SK-RILs were divided into two groups, one group with allele from SX828 and the other with allele from KN2007, to perform mean comparison regarding TKW. The positive allele of *qTKW-5B.2* from SX828 increased TKW value of 2.1–3.5 g, indicating a tremendous potential for its application in wheat molecular breeding programs designed to increase grain output (**Supplementary Figure S6**).

### Other Important Clusters Associated With Kernel Traits

Genes and QTL for multiple kernel shape and size are usually linked or show pleiotropic effects. Several genetic loci simultaneously controlling KW and TKW were identified in the C1 on chromosome 1A in our study (**Table 4**). This result is in consistence with the report of Ramya et al. (2010), who detected a QTL controlling TKW between markers *Xwmc24* and *Xgwm357* in the same genetic region.

Cluster 4, simultaneously facilitated KW and TKW, was mapped on chromosome 3A, and most likely corresponds to *TaGS5* (Ma et al., 2016). *TaGS5*, an ortholog of rice grain size gene *OsGS5*, is associated with kernel width and kernel weight in wheat. *OsGS5* which encodes a putative serine carboxypeptidase, promotes cell division by regulating cell cycle genes resulting in large grain size generated by an increased cell number (Li et al., 2011). The high homology of *OsGS5* and *TaGS5-3A* suggests a similar function in wheat.

QTL for KW and TKW were co-located in cluster 5 on chromosome 4B. The same genomic region was found associated with QTL for KL, KW and TKW in the Chinese winter wheat line Yanda1817 (Wu et al., 2015). Kumar et al. (2016) also identified a QTL (QTL-15) in the same region related to KW, KA (kernel area) and TKW, and proposed this genomic region could harbor an ortholog of rice gene *GS3* (Huang et al., 2013) which encodes a putative protein phosphatase with a Kelch-like repeat domain (OsPPKL1) and has positive effect on kernel size and weight (Zhang X. et al., 2012). Thus, this region is noteworthy for genetic improvement of kernel weight and size in wheat.

## Conclusion

A high-density genetic linkage map was constructed for the Shixin828/Kenong2007 RIL population using the iSelect 90K SNP array, which is in good accordance with the recently released CS wheat genome assembly. This genetic map was proved powerful for mapping QTL of kernel size and weight. As results, two important QTL clusters on chromosomes 2D and 5B that are associated with TKW and kernel size were identified by unconditional and conditional analyses. These QTL clusters could serve as target regions for fine mapping and MAS in wheat breeding.

## Author Contributions

QS, WZ, and JuL designed the research. QS, WZ, LL, and XlZ conducted genotyping of the SK-RIL population. QS, WZ, XlZ, LL, LZ, NZ, LS, XX, GL, JjL, DM, JJ, XuZ, CY, YT, and JmL conducted phenotyping of the SK-RIL population. QS, XlZ, NZ, WZ, ZL, and JuL analyzed the data and wrote the paper. WZ and JuL had primary responsibility for final content. All authors read and approved the final manuscript.

## Conflict of Interest Statement

The authors declare that the research was conducted in the absence of any commercial or financial relationships that could be construed as a potential conflict of interest.

## Footnotes

1 http://hzwseen.foodmate.net/

2 http://en.wikipedia.org/wiki/SPSS

3 http://ibi.zju.edu.cn/software/qga/

4 http://www.capitalbio.com

5 http://www.isbreeding.net

6 http://www.biometris.nl/uk/Sofware/MapChart/

7 https://urgi.versailles.inra.fr/download/iwgsc/IWGSC_RefSeq_Assemblies/v1.0/
