# Supplementary figures and images for: QTL Detection for Kernel Size and Weight in Bread Wheat (Triticum aestivum L.) Using a High-Density SNP and SSR-Based Linkage Map
Source: Front Plant Sci. 2018 Oct 11;9:1484. doi: 10.3389/fpls.2018.01484 (PMC6193082; doi:10.3389/fpls.2018.01484)

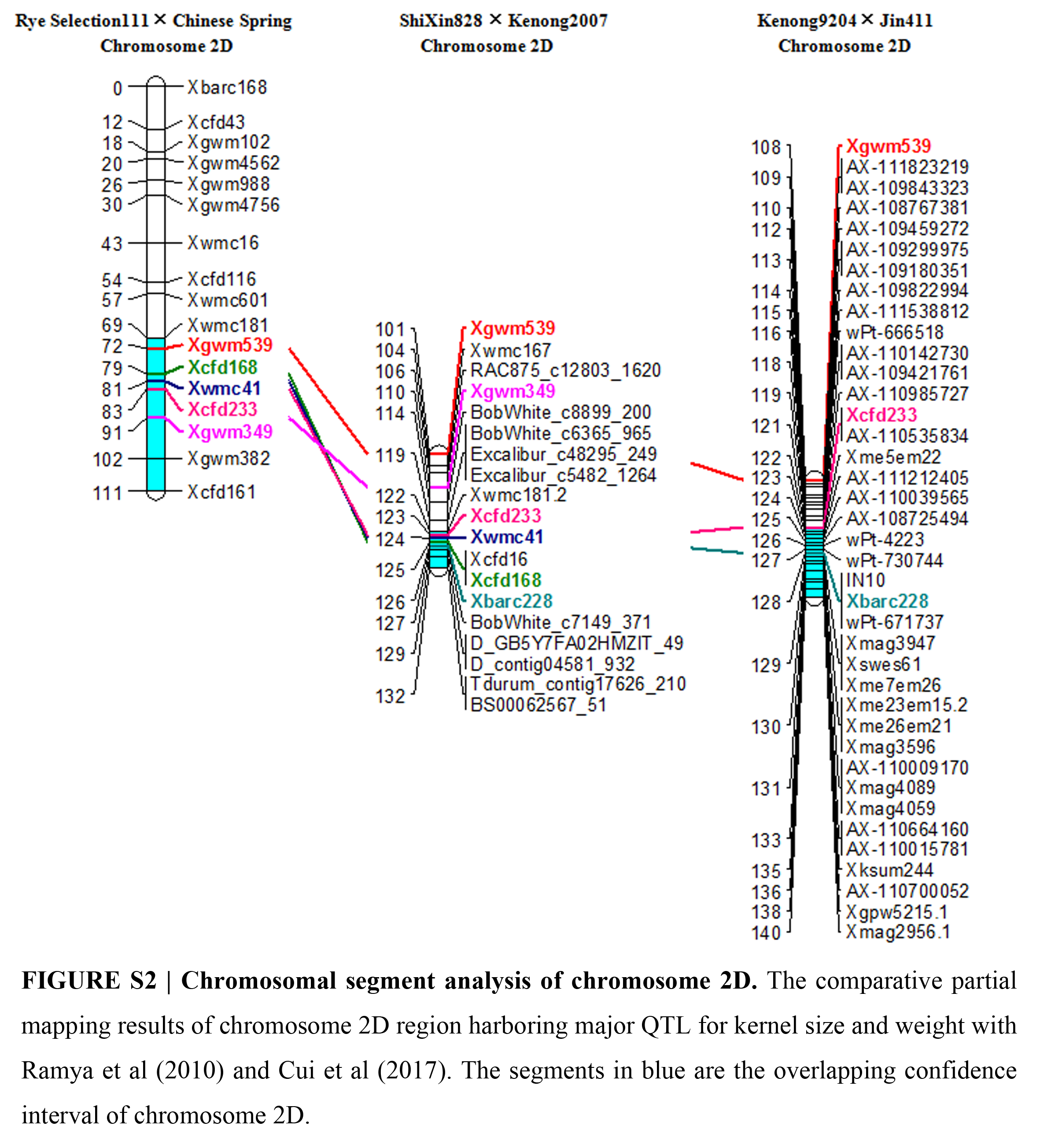

Supplement: Supplementary file 1 [file Image_1.TIF]

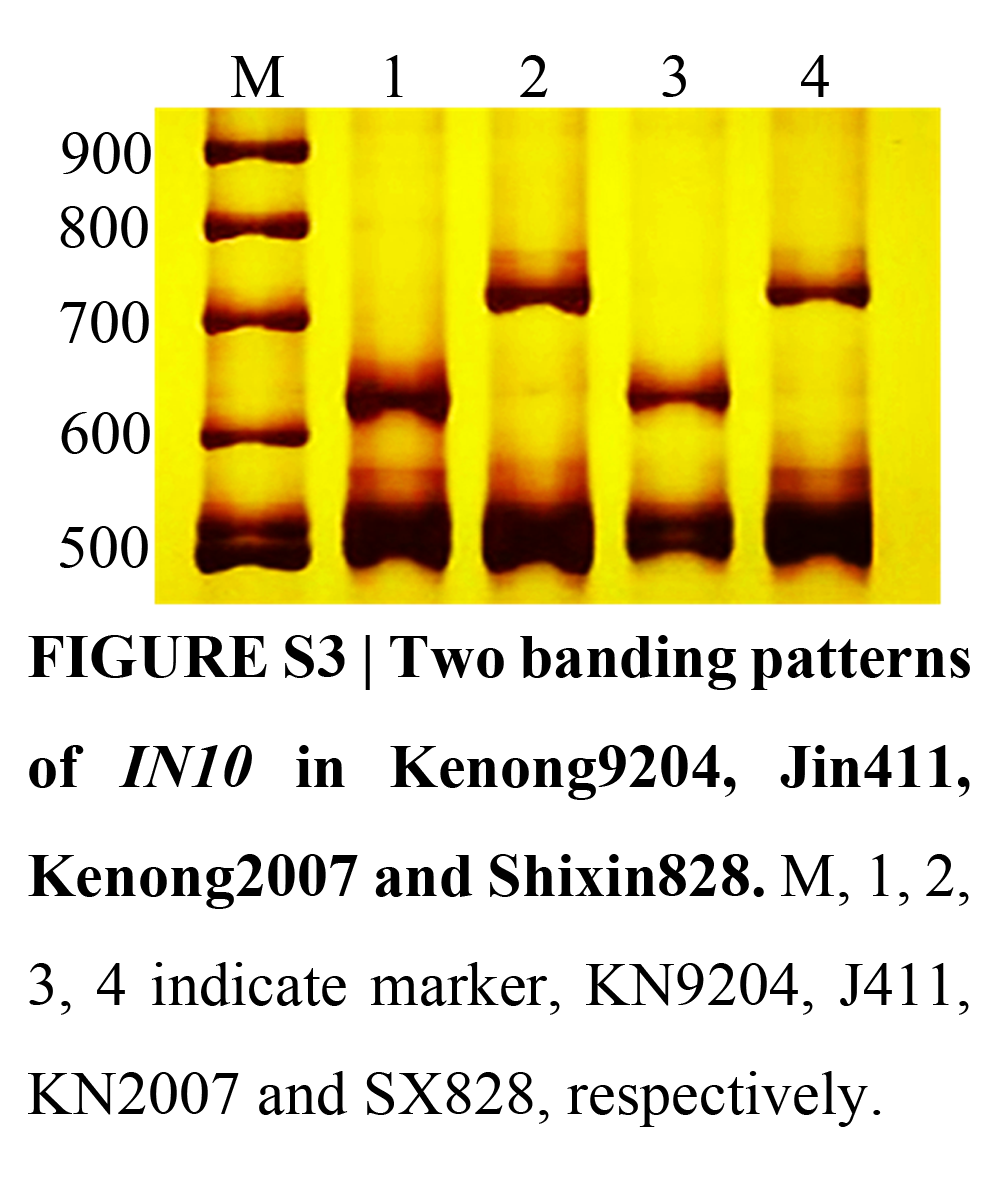

Supplement: Supplementary file 2 [file Image_2.TIF]

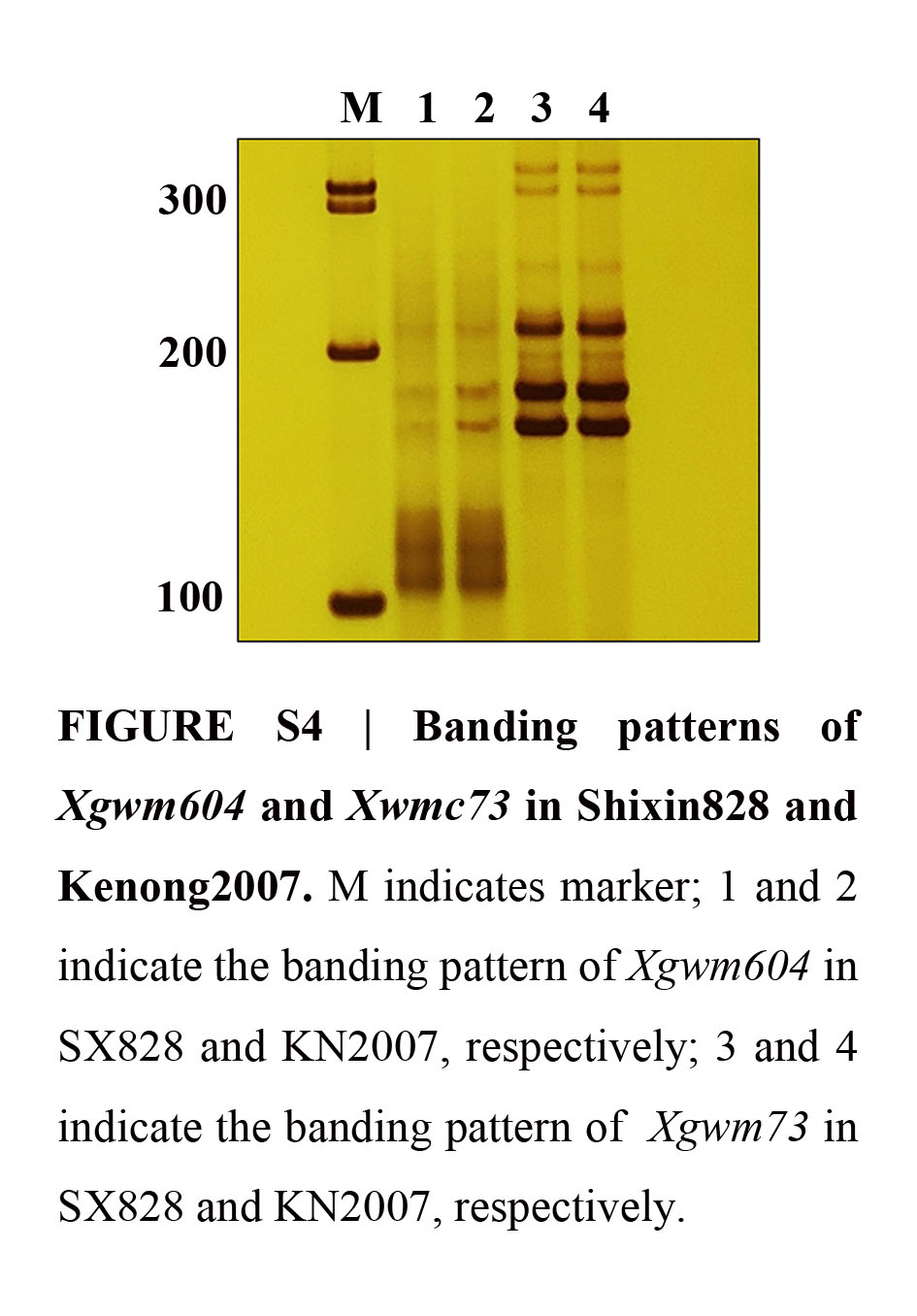

Supplement: Supplementary file 3 [file Image_3.TIF]

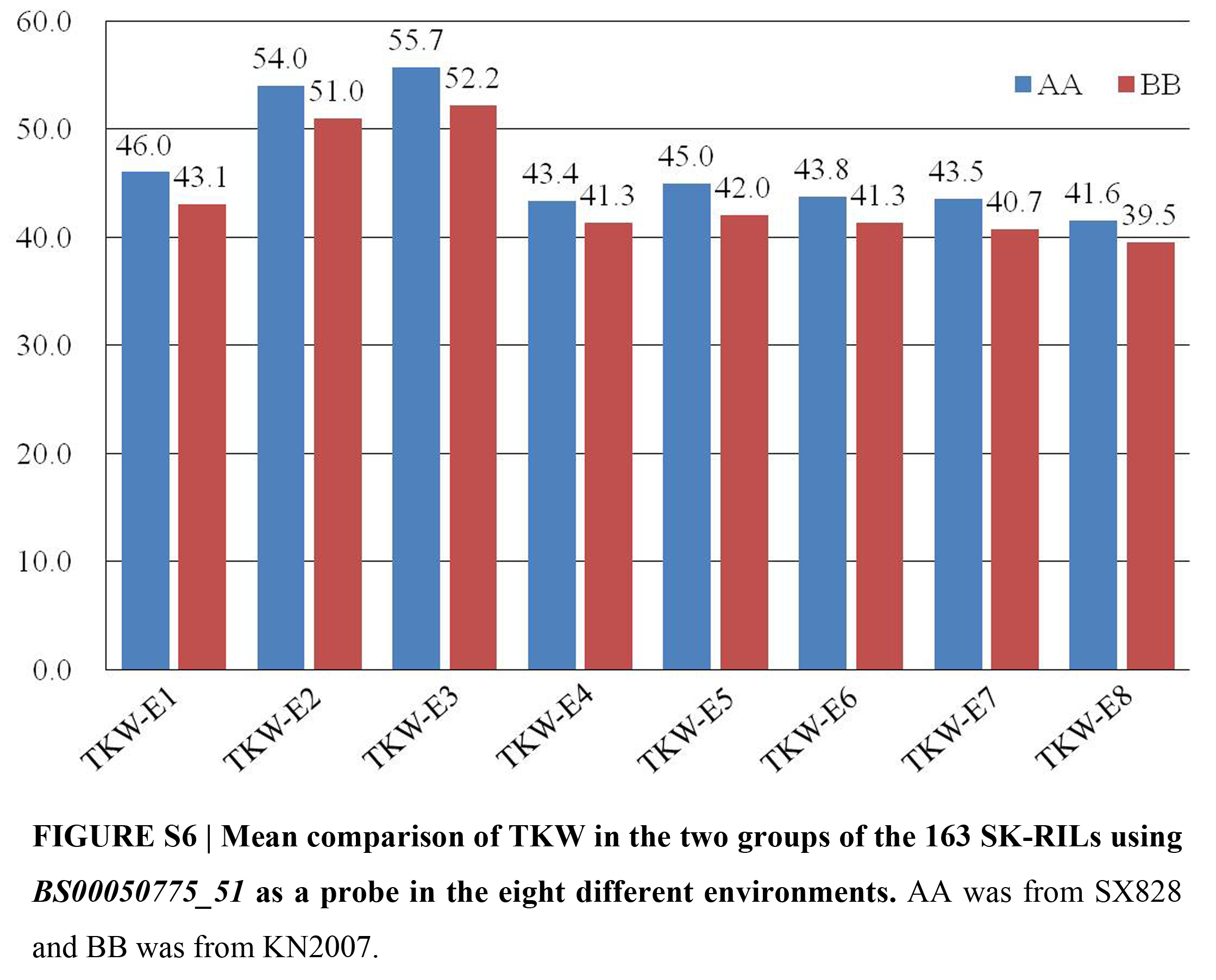

Supplement: Supplementary file 4 [file Image_4.TIF]
