# Supplementary material for: QTL Detection for Kernel Size and Weight in Bread Wheat (Triticum aestivum L.) Using a High-Density SNP and SSR-Based Linkage Map
Source: Front Plant Sci. 2018 Oct 11;9:1484. doi: 10.3389/fpls.2018.01484 (PMC6193082; doi:10.3389/fpls.2018.01484)
Supplement: Supplementary file 5 [file Table_1.DOCX]

**Table S1** Summary of the year, location and nitrogen treatment conditions.

| En.^a^ | Year | Location | Soli nitrate-N contents  (mg kg^-1^) | Soli total-N contents  (mg kg^-1^) |
| --- | --- | --- | --- | --- |
| E1-HN | 2014-2015 | Shijiazhuang | 45.2 | 122.5 |
| E2-HN | 2015-2016 | Shijiazhuang | 46.3 | 125.4 |
| E3-LN | 2015-2016 | Shijiazhuang | 15.6 | 81.8 |
| E4-HN | 2016-2017 | Shijiazhuang | 43.5 | 124.6 |
| E5-LN | 2016-2017 | Shijiazhuang | 14.5 | 80.5 |
| E6-HN | 2016-2017 | Anyang | 36.7 | 121.7 |
| E7-HN | 2016-2017 | Beijing | 39.7 | 124.3 |
| E8-LN | 2016-2017 | Beijing | 20.9 | 88.3 |

^a^ En. = environments.
