# Supplementary material for: QTL Detection for Kernel Size and Weight in Bread Wheat (Triticum aestivum L.) Using a High-Density SNP and SSR-Based Linkage Map
Source: Front Plant Sci. 2018 Oct 11;9:1484. doi: 10.3389/fpls.2018.01484 (PMC6193082; doi:10.3389/fpls.2018.01484)
Supplement: Supplementary file 8 [file Table_4.DOCX]

**Table S4** Correlation coefficients between yield-related traits in eight environments.

| Traits ^a^ | KL | KW | KDR | TKW | SNPP | KNPS |
| --- | --- | --- | --- | --- | --- | --- |
| KL | 1 |  |  |  |  |  |
| KW | -0.014 | 1 |  |  |  |  |
| KDR | 0.854^**^ | -0.529^**^ | 1 |  |  |  |
| TKW | 0.592^**^ | 0.696^**^ | 0.134 | 1 |  |  |
| SNPP | 0.107 | -0.304^**^ | 0.248^**^ | -0.115 | 1 |  |
| KNPS | -0.166^*^ | -0.096 | -0.091 | -0.313^**^ | -0.275^**^ | 1 |

^a^ The average phenotypic value of the traits in the eight environments was used to conduct the correlation analysis.

^*^Significant at *P* < 0.05 level.

^**^Significant at *P* < 0.01 level.
