# Supplementary material for: QTL Detection for Kernel Size and Weight in Bread Wheat (Triticum aestivum L.) Using a High-Density SNP and SSR-Based Linkage Map
Source: Front Plant Sci. 2018 Oct 11;9:1484. doi: 10.3389/fpls.2018.01484 (PMC6193082; doi:10.3389/fpls.2018.01484)
Supplement: Supplementary file 9 [file Table_5.DOCX]

**Table S5** Unconditional QTL with significant additive effects for TKW and kernel size in SK-RIL population.

| Traits | QTL ^a^ | Leaf markers | Right markers | Environments ^b^ | LOD value | | PVE% ^c^ | | Add ^d^ | |
| --- | --- | --- | --- | --- | --- | --- | --- | --- | --- | --- |
|  |  |  |  |  | Range | Mean | Range | Mean | Range | Mean |
| KL | *qKL-1A* | *Wp_Ex_rep_c67665_66326492* | *Bb_c44224_71* | E2 | 3.10 | 3.10 | 4.20 | 4.20 | -0.08 | -0.08 |
|  | *qKL-1D* | *Wp_Ex_c35886_43950102* | *Exb_c50856_63* | E6/E7 | 3.21/4.33 | 3.77 | 4.63/6.70 | 5.67 | 0.07/0.08 | 0.07 |
|  | *qKL-2A* | *Kukri_c9118_1891* | *RAC875_c10218_91* | E2 | 3.40 | 3.40 | 4.74 | 4.74 | 0.07 | 0.07 |
|  | ***qKL-2B.1*** | *RAC875_rep_c109207_706* | *Wp_JD_c20720_18369335* | E1 | 8.20 | 8.20 | 11.53 | 11.53 | -0.10 | -0.10 |
|  | *qKL-2B.2* | *Td_cg41990_1267* | *BS00082137_51* | E2/E3/E4 | 5.25/4.45/3.25 | 4.32 | 7.73/7.69/5.00 | 6.81 | -0.09/-0.09/-0.07 | -0.09 |
|  | *qKL-2B.3* | *BS00012081_51* | *Td_cg13432_860* | E4/E8 | 2.69/3.17 | 2.93 | 5.28/9.99 | 7.63 | -0.07/-0.10 | -0.09 |
|  | *qKL-2D* | *Xwmc41* | *BS00062567_51* | E1/E3/E4/  E5/E7/E8 | 3.62/2.27/4.43/  9.92/3.65/6.48 | 5.14 | 4.58/4.27/6.88/  10.14/5.24/9.85 | 6.83 | 0.06/0.07/0.08/  0.10/0.07/0.10 | 0.08 |
|  | *qKL-3A.1* | *Exb_rep_c109598_66* | *Wp_Ku_c217_430915* | E1/E2/E3 | 4.49/2.86/2.83 | 3.54 | 6.43/7.05/6.80 | 6.76 | 0.08/0.09/0.08 | 0.08 |
|  | *qKL-3A.2* | *RAC875_c38647_290* | *CAP11_rep_c8579_223* | E3/E6 | 3.08/2.86 | 2.97 | 7.70/6.62 | 7.16 | 0.09/0.08 | 0.09 |
|  | *qKL-3B* | *IAAV5302* | *Exb_c35203_334* | E6 | 5.92 | 5.92 | 8.81 | 8.81 | 0.09 | 0.09 |
|  | *qKL-4A* | *RAC875_c10748_532* | *Xwmc258-4A_5B* | E6 | 2.67 | 2.67 | 7.32 | 7.32 | -0.09 | -0.09 |
|  | ***qKL-5B.1*** | *Wp_Ku_c14332_22613741* | *Exb_c631_219* | E2/E4/E7 | 11.33/9.83/10.62 | 10.59 | 17.04/15.86/16.79 | 16.56 | 0.14/0.13/0.13 | 0.13 |
|  | *qKL-5B.2* | *Ku_c1083_1314* | *BS00022477_51* | E1/E5 | 5.35/5.10 | 5.23 | 7.24/4.65 | 5.95 | 0.08/0.07 | 0.08 |
|  | *qKL-5B.3* | *Wp_Ex_rep_c68017_66762485* | *IACX751* | E4 | 3.22 | 3.22 | 5.33 | 5.33 | 0.08 | 0.08 |
|  | *qKL-6B.1* | *BS00011479_51* | *BS00022832_51* | E4 | 5.52 | 5.52 | 9.51 | 9.51 | 0.10 | 0.10 |
|  | *qKL-6B.2* | *BS00011202_51* | *RAC875_s109956_75* | E1/E2/E3/  E6/E7 | 6.76/7.05/6.60/  5.84/3.38 | 5.93 | 12.25/10.11/11.12/  8.77/4.89 | 9.43 | 0.11/0.10/0.11/  0.09/0.07 | 0.10 |
|  | *qKL-7A* | *BS00062824_51* | *Wp_Ex_c53019_56399917* | E5 | 3.28 | 3.28 | 4.14 | 4.14 | 0.07 | 0.07 |
|  | *qKL-7B* | *Wp_Ex_c45195_51056529* | *Kukri_c50071_1084* | E8 | 3.65 | 3.65 | 5.44 | 5.44 | 0.07 | 0.07 |
| KW | ***qKW-1A.1*** | *Wp_CAP12_c2438_1180601* | *Td_cg8382_300* | E4 | 6.86 | 6.86 | 13.80 | 13.80 | -0.05 | -0.05 |
|  | *qKW-1A.2* | *Xgwm164* | *Xwmc278-1A* | E2 | 5.23 | 5.23 | 9.54 | 9.54 | -0.04 | -0.04 |
|  | *qKW-1D* | *BS00003816_51* | *Wp_Ex_c6378_11087794* | E1 | 2.90 | 2.90 | 5.93 | 5.93 | -0.04 | -0.04 |
|  | ***qKW-2D.1*** | *Xwmc181.2* | *D_GB5Y7FA02HMZIT_49* | E1/E2/E3/  E5/E6/  E7/E8 | 3.59/5.20/6.57/  10.07/5.08/  10.25/6.03 | 6.68 | 7.55/9.34/13.54/  12.52/7.01/  18.82/16.29 | 12.15 | -0.04/-0.04/-0.04/  -0.04/-0.04/  -0.05/-0.05 | -0.04 |
|  | ***qKW-2D.2*** | *RAC875_c6144_121* | *tplb0049b24_1152* | E1 | 5.02 | 5.02 | 12.77 | 12.77 | 0.06 | 0.06 |
|  | *qKW-3A* | *Exb_c32653_553* | *RFL_cg1896_1236* | E5 | 4.53 | 4.53 | 5.47 | 5.47 | -0.03 | -0.03 |
|  | *qKW-3B.1* | *BS00010332_51* | *GENE-2732_162* | E2 | 4.41 | 4.41 | 7.94 | 7.94 | 0.04 | 0.04 |
|  | *qKW-3B.2* | *Kukri_c16742_415* | *IAAV7128* | E3 | 2.98 | 2.98 | 5.93 | 5.93 | 0.03 | 0.03 |
|  | ***qKW-4A*** | *Ku_c6779_1381* | *Xwmc258* | E6 | 8.51 | 8.51 | 15.51 | 15.51 | -0.06 | -0.06 |
|  | *qKW-4B.1* | *BS00068104_51* | *Kukri_c52413_282* | E4/E5/E6 | 4.28/4.81/3.92 | 4.34 | 7.98/6.16/5.88 | 6.67 | 0.04/0.03/0.04 | 0.03 |
|  | *qKW-4B.2* | *RFL_cg3363_1294* | *Td_cg56458_594* | E7 | 4.31 | 4.31 | 8.52 | 8.52 | 0.03 | 0.03 |
|  | *qKW-4B.3* | *tplb0056b15_858* | *Wp_BE403378B_Ta_2_1* | E5 | 3.98 | 3.98 | 4.63 | 4.63 | 0.02 | 0.02 |
|  | *qKW-5A* | *Xwmc631* | *Wp_Ex_c4211_7606269* | E5 | 2.77 | 2.77 | 6.17 | 6.17 | -0.03 | -0.03 |
|  | *qKW-5B* | *CAP7_c5481_96* | *Xwmc386* | E5/E6/E7 | 3.54/2.53/3.37 | 3.14 | 4.85/3.59/6.43 | 4.96 | 0.02/0.03/0.03 | 0.03 |
|  | *qKW-5D* | *D_GA8KES402JRBN7_105* | *GENE-3619_813* | E2 | 3.17 | 3.17 | 5.60 | 5.60 | 0.03 | 0.03 |
|  | *qKW-6A.1* | *Xwmc773* | *Xgwm617* | E3 | 2.66 | 2.66 | 5.21 | 5.21 | -0.03 | -0.03 |
|  | *qKW-6A.2* | *Wp_Ra_c11651_18855691* | *CAP8_c1237_68* | E7 | 2.82 | 2.82 | 4.80 | 4.80 | -0.03 | -0.03 |
|  | *qKW-6D* | *Td_cg12045_1230* | *GENE-4225_117* | E7 | 2.60 | 2.60 | 6.74 | 6.74 | -0.03 | -0.03 |
|  | *qKW-7B.1* | *Bb_c39364_231* | *Wp_Ex_c9909_16316813* | E4 | 3.81 | 3.81 | 6.94 | 6.94 | -0.03 | -0.03 |
|  | *qKW-7B.2* | *Xgwm302* | *Wp_Ex_c45195_51056529* | E6 | 5.92 | 5.92 | 8.77 | 8.77 | -0.05 | -0.05 |
| KDR | *qKDR-1D.1* | *D_cg32652_163* | *Xcfd65* | E2 | 3.44 | 3.44 | 7.09 | 7.09 | -0.03 | -0.03 |
|  | *qKDR-1D.2* | *BS00003816_51* | *Wp_Ex_c35886_43949442* | E1/E4 | 6.45/5.09 | 5.77 | 10.70/5.92 | 8.31 | 0.03/0.03 | 0.03 |
|  | *qKDR-1D.3* | *BS00064881_51* | *Xcfd63* | E7/E8 | 3.12/4.25 | 3.68 | 6.49/8.30 | 7.40 | 0.03/0.03 | 0.03 |
|  | *qKDR-2B.1* | *RFL_cg4194_1665* | *Wp_Ex_c7003_12065567* | E2/E3 | 3.72/4.01 | 3.87 | 4.45/5.82 | 5.13 | -0.02/-0.02 | -0.02 |
|  | *qKDR-2B.2* | *Kukri_c9898_1766* | *Xwmc661.1* | E2/E3 | 3.97/3.44 | 3.71 | 5.14/5.03 | 5.09 | 0.02/0.02 | 0.02 |
|  | ***qKDR-2D.1*** | *Xcfd168* | *Bb_c7149_371* | E1/E2/E3/  E4/E5/E6  /E7/E8 | 7.83/12.47/12.01/  11.70/12.81/7.88/  8.11/8.93 | 10.22 | 13.78/16.39/18.22/  15.54/16.07/17.27/  17.15/18.10 | 16.57 | 0.04/0.04/0.04/  0.04/0.04/0.04/  0.05/0.05 | 0.04 |
|  | *qKDR-2D.2* | *RAC875_rep_c111100_447* | *Bb_rep_c49468_443* | E5 | 5.68 | 5.68 | 6.11 | 6.11 | 0.03 | 0.03 |
|  | *qKDR-3A* | *Wp_Ku_c5359_9530161* | *Td_cg48522_295* | E1 | 3.13 | 3.13 | 5.86 | 5.86 | 0.03 | 0.03 |
|  | *qKDR-4A.1* | *Kukri_c9259_678* | *BS00108849_51* | E6/E8 | 2.71/2.58 | 2.65 | 7.62/6.85 | 7.23 | 0.03/0.03 | 0.03 |
|  | ***qKDR-4A.2*** | *Kukri_c9259_421* | *Kukri_c31087_82* | E4 | 6.79 | 6.79 | 10.27 | 10.27 | 0.03 | 0.03 |
|  | *qKDR-5A.1* | *BS00034303_51* | *Td_cg13810_485* | E4 | 2.67 | 2.67 | 4.17 | 4.17 | 0.02 | 0.02 |
|  | ***qKDR-5A.2*** | *Xwmc631* | *Wp_Ex_c4211_7606269* | E5 | 7.35 | 7.35 | 14.39 | 14.39 | 0.04 | 0.04 |
|  | *qKDR-5A.3* | *Xgwm291* | *Xwmc74* | E5 | 4.25 | 4.25 | 4.61 | 4.61 | 0.02 | 0.02 |
|  | *qKDR-5B.1* | *Xgwm540* | *Xwmc386* | E1/E8 | 3.72/2.88 | 3.30 | 7.45/5.23 | 6.34 | 0.03/0.03 | 0.03 |
|  | *qKDR-5B.2* | *BS00050775_51* | *Exb_c37146_747* | E4/E5 | 7.75/4.09 | 5.92 | 9.26/5.20 | 7.23 | 0.03/0.02 | 0.03 |
|  | *qKDR-6A.1* | *Xwmc684* | *Kukri_c21296_217* | E4 | 4.44 | 4.44 | 6.11 | 6.11 | -0.03 | -0.03 |
|  | *qKDR-6A.2* | *Bb_c10832_1131* | *Wp_JD_rep_c65886_41872083* | E3 | 3.15 | 3.15 | 4.61 | 4.61 | 0.02 | 0.02 |
|  | ***qKDR-6A.3*** | *Xwmc621* | *Ra_c6429_1217* | E4/E6 | 9.59/5.62 | 7.60 | 12.65/19.06 | 15.86 | 0.04/0.04 | 0.04 |
|  | *qKDR-6B* | *BS00011202_51* | *TA005327-0480* | E2/E3/E7 | 6.28/4.33/3.93 | 4.84 | 7.63/5.99/7.98 | 7.20 | 0.03/0.02/0.03 | 0.03 |
| TKW | *qTKW-1A.1* | *Wp_CAP12_c2438_1180601* | *Ex_c2389_1834* | E6/E7 | 2.70/2.91 | 2.81 | 5.15/3.96 | 4.56 | -0.85/-0.71 | -0.78 |
|  | *qTKW-1A.2* | *Xgwm164* | *Xgwm135* | E2/E4 | 2.92/4.95 | 3.94 | 5.37/9.54 | 7.45 | -1.02/-1.24 | -1.13 |
|  | *qTKW-1B* | *Wp_Ex_c3147_5816957* | *Td_cg10362_555* | E3 | 3.56 | 3.56 | 5.46 | 5.46 | 0.99 | 0.99 |
|  | *qTKW-2A.1* | *Xgwm356* | *Wp_Ex_c14953_23104041* | E2/E5 | 2.54/2.91 | 2.72 | 3.73/6.05 | 4.89 | 0.84/0.80 | 0.82 |
|  | *qTKW-2A.2* | *GENE-1080_39* | *RAC875_c39634_370* | E1 | 3.54 | 3.54 | 8.94 | 8.94 | -1.48 | -1.48 |
|  | ***qTKW-2B*** | *TA005292-0114* | *Kukri_rep_c109142_264* | E7/E8 | 5.10/5.86 | 5.48 | 8.68/14.92 | 11.80 | -1.04/-1.44 | -1.24 |
|  | ***qTKW-2D.1*** | *D_cg02226_528* | *Bb_c16655_421* | E6 | 4.60 | 4.60 | 10.64 | 10.64 | 1.21 | 1.21 |
|  | *qTKW-2D.2* | *Kukri_c74165_204* | *GENE-0638_1278* | E3 | 4.24 | 4.24 | 8.12 | 8.12 | -1.24 | -1.24 |
|  | *qTKW-2D.3* | *RAC875_c6144_121* | *tplb0049b24_1152* | E1 | 2.90 | 2.90 | 7.16 | 7.16 | 1.47 | 1.47 |
|  | ***qTKW-3A.1*** | *Exb_c32653_553* | *RFL_cg1896_1236* | E7 | 9.67 | 9.67 | 13.74 | 13.74 | -1.38 | -1.38 |
|  | *qTKW-3A.2* | *Wp_Ku_c5359_9530161* | *Td_cg48522_295* | E3 | 3.21 | 3.21 | 5.53 | 5.53 | 1.01 | 1.01 |
|  | *qTKW-3A.3* | *Wp_Ku_c7811_13387117* | *CAP11_c1022_117* | E2/E5 | 5.34/2.51 | 3.92 | 8.08/3.49 | 5.78 | 1.24/0.60 | 0.92 |
|  | ***qTKW-3B*** | *Wp_Ex_c11893_19077166* | *Wp_Ex_rep_c66893_65301351* | E3/E5 | 8.07/6.25 | 7.16 | 13.02/9.05 | 11.03 | 1.50/0.97 | 1.24 |
|  | *qTKW-4B* | *Xwmc710* | *IACX938* | E5 | 2.63 | 2.63 | 3.95 | 3.95 | 0.64 | 0.64 |
|  | *qTKW-5A* | *RAC875_c58966_471* | *Wp_Ex_c2474_4619730* | E1/E2/E4/E6 | 2.57/5.34/2.57/2.54 | 3.26 | 5.30/8.07/4.80/4.60 | 5.70 | 1.14/1.25/0.88/0.78 | 1.01 |
|  | *qTKW-5B.1* | *Xwmc740* | *Xwmc386* | E7/E8 | 5.13/4.41 | 4.77 | 7.92/10.27 | 9.09 | 0.99/1.24 | 1.11 |
|  | ***qTKW-5B.2*** | *BS00050775_51* | *Exb_c631_219* | E2/E4/E5/  E6/E7 | 9.34/5.68/15.05/  5.94/3.01 | 9.00 | 14.72/11.12/24.41/  11.13/10.04 | 15.34 | 1.68/1.34/1.60/  1.21/1.15 | 1.46 |
|  | *qTKW-5B.3* | *RFL_cg4167_1164* | *Xcfd7* | E8 | 2.61 | 2.61 | 6.39 | 6.39 | 0.95 | 0.95 |
|  | *qTKW-6A* | *Exb_c431_1130* | *BS00065069_51* | E2 | 3.42 | 3.42 | 5.35 | 5.35 | -1.01 | -1.01 |
|  | ***qTKW-6D*** | *Wp_Ex_rep_c68175_66950470* | *tplb0057e06_1607* | E5/E8 | 5.56/4.13 | 4.84 | 9.63/10.77 | 10.20 | -1.00/-1.22 | -1.11 |
|  | *qTKW-7A* | *Wp_Ku_rep_c110993_94857161* | *RAC875_c22792_672* | E7 | 5.26 | 5.26 | 7.44 | 7.44 | -0.97 | -0.97 |

^a^ A putative major QTL is marked by bold typeface which characterized by a mean LOD > 3.0 and a mean PVE >10 %; a putative stable QTL is underlined when this locus can be detected in at least four of the eight environments.

^b^ E1, E2, E3, E4, E5, E6, E7 and E8 indicate the trails were conducted in: 2014-2015, Shijiazhuang, high nitrogen (HN); 2015-2016, Shijiazhuang, HN; 2015-2016, Shijiazhuang, low nitrogen (LN); 2016-2017, Shijiazhuang, HN; 2016-2017, Shijiazhuang, LN; 2016-2017, Anyang, HN; 2016-2017, Beijing, HN and 2016-2017, Beijing, LN, respectively.

^c^ PVE indicates the percentage of explained phenotypic variation.

^d^ A positive sign means that the positive alleles come from the parent SX828; a negative sign means that the positive alleles come from the parent KN2007.
