# Supplementary material for: QTL Detection for Kernel Size and Weight in Bread Wheat (Triticum aestivum L.) Using a High-Density SNP and SSR-Based Linkage Map
Source: Front Plant Sci. 2018 Oct 11;9:1484. doi: 10.3389/fpls.2018.01484 (PMC6193082; doi:10.3389/fpls.2018.01484)
Supplement: Supplementary file 11 [file Table_7.DOCX]

**Table S7 Partial stable QTL for TKW and kernel size on HN and LN conditions**

| **QTL** | **En ^a^** | **LOD ^b^** | **PVE % ^c^** | **Add ^d^** | **HN** | | | | |  | **LN** | | | | |
| --- | --- | --- | --- | --- | --- | --- | --- | --- | --- | --- | --- | --- | --- | --- | --- |
|  |  |  |  |  | **LOD**  **(A) ^e^** | **LOD**  **(AbyE) ^f^** | **PVE**  **(A) % ^g^** | **PVE**  **(AbyE) % ^h^** | **Add**  **(A) ^i^** |  | **LOD**  **(A)** | **LOD**  **(AbyE)** | **PVE**  **(A) %** | **PVE**  **(AbyE) %** | **Add**  **(A)** |
| *qKL-2D* | E1/E3/E4/  E5/E7/E8 | 2.27-9.92 | 4.27-10.14 | 0.06 to 0.10 | 8.72 | 5.69 | 2.18 | 1.37 | 0.05 |  | 2.63 | 1.97 | 1.25 | 0.88 | 0.04 |
| *qKL-6B.2* | E1/E2/E3/  E6/E7 | 3.38-7.05 | 4.89-12.25 | 0.07 to 0.11 | 22.81 | 4.08 | 5.84 | 1.32 | 0.08 |  | 4.42 | 3.03 | 2.10 | 2.19 | 0.05 |
| ***qKW-2D.1*** | E1/E2/E3/E5/  E6/E7/E8 | 3.59-10.25 | 7.01-18.82 | -0.04 to -0.05 | 8.78 | 5.60 | 2.85 | 1.70 | -0.02 |  | 6.43 | 6.45 | 3.46 | 2.65 | -0.02 |
| ***qKDR-2D.1*** | E1/E2/E3/E4/  E5/E6/E7/E8 | 7.83-12.81 | 13.78-18.22 | 0.04 to 0.05 | 39.53 | 0.65 | 14.51 | 0.11 | 0.04 |  | 27.65 | 0.91 | 15.50 | 0.08 | 0.04 |
| *qTKW-5A* | E1/E2/E4/E6 | 2.54-5.34 | 4.60-8.07 | 0.78 to 1.25 | 17.09 | 6.21 | 5.80 | 1.55 | 0.99 |  | 1.39 | 1.57 | 0.70 | 0.61 | 0.31 |
| ***qTKW-5B.2*** | E2/E4/E5/  E6/E7 | 3.01-15.05 | 10.04-24.41 | 1.15 to 1.68 | 7.04 | 10.96 | 2.34 | 2.96 | 0.63 |  | 4.20 | 9.24 | 2.22 | 3.15 | 0.56 |

^a^ E1, E2, E3, E4, E5, E6, E7 and E8 indicate trial 1 (2014–2015, Shijiazhuang) HN, trial 2 (2015–2016, Shijiazhuang) HN, trial 2 LN, trial 3 (2016–2017, Shijiazhuang) HN, trial 3 LN, trial 4 (2016–2017, Anyang) HN, trial 5 (2016–2017, Beijing) HN, and trial 5 LN, respectively.

^b, c, d^ Values of the corresponding additive QTL in individual environment. A positive sign indicates alleles from SX828 increased the corresponding trait value and a negative sign indicates alleles from KN2007 increased the corresponding trait value. PVE, phenotypic variance explanation; Add, additive effect.

^e, g, i^ Values of the corresponding additive QTL in combined QTL analysis across environments.

^f, h^ Values for interaction effect of A (additive) by E (environment) of the corresponding additive QTL in combined QTL analysis across environments.
