# Supplementary material for: QTL Detection for Kernel Size and Weight in Bread Wheat (Triticum aestivum L.) Using a High-Density SNP and SSR-Based Linkage Map
Source: Front Plant Sci. 2018 Oct 11;9:1484. doi: 10.3389/fpls.2018.01484 (PMC6193082; doi:10.3389/fpls.2018.01484)
Supplement: Supplementary file 12 [file Table_8.DOCX]

**Table S8 Conditional QTL for TKW with respect to SNPP and KNPS.**

| Cluster ^a^ | QTL ^b^ | Interval markers ^c^ | Unconditional QTL | Conditional QTL | |
| --- | --- | --- | --- | --- | --- |
|  |  |  | Additive [En/PVE(%)] ^d^ | Additive [En/PVE(%)] | |
|  |  |  | TKW | TKW\|SNPP | TKW\|KNPS |
| C1 | *qTKW-1A.1* | *Wp_CAP12_c2438_1180601*—*Ex_c2389_1834* |  | -1.08(E4/6.17) | -1.16(E4/8.41) |
|  |  |  | -0.85(E6/5.15) |  |  |
|  |  |  | -0.71(E7/3.96) |  |  |
| C2 | *qTKW-1A.2* | *Xgwm164—Xgwm135* | -1.02(E2/5.37) |  | -1.07(E2/6.21) = |
|  |  |  | -1.24(E4/9.54) |  |  |
|  |  |  |  | -0.83(E7/5.52) |  |
| C3 | *qTKW-2D.4* | *Xwmc181.2—BS00062567_51* |  |  |  |
| C4 | *qTKW-3A.1* | *Exb_c32653_553—RFL_cg1896_1236* | -1.38(E7/13.74) | -1.25(E7/12.30) = | -1.38(E7/16.17) = |
| C5 | *qTKW-4B.1* | *BS00068104_51—Kukri_c52413_282* | 0.64(E5/3.95) | 0.99(E5/9.43) + |  |
| C6 | *qTKW-5B.1* | *CAP7_c5481_96—Xwmc386* | 0.99(E7/7.92) | 1.02(E7/8.54) = | 1.64(E7/23.87) + |
|  |  |  | 1.24(E8/10.27) |  |  |
| C7 | ***qTKW-5B.2*** | *BS00050775_51—Exb_c37146_747* | 1.68(E2/14.72) | 1.56(E2/12.65) = | 1.62(E2/14.16) = |
|  |  |  | 1.34(E4/11.12) | 1.47(E4/13.34) = | 1.58(E4/15.75) + |
|  |  |  | 1.60(E5/24.41) | 1.51(E5/21.49) = | 1.35(E5/17.49) - |
|  |  |  | 1.21(E6/11.13) | 1.26(E6/12.30) = |  |
|  |  |  | 1.15(E7/10.04) |  |  |
|  |  |  |  | 0.89(E8/5.79) | 0.91(E8/6.56) |

^a^ QTL for kernel-related traits located in the 7 clusters in Table 4. A putative major QTL is marked by bold typeface which is characterized by a mean LOD value >3.0 and a mean PVE>10 %; a putative stable QTL is underlined when this locus can be detected in at least four of the eight environments.

^b^ The clusters containing QTL affecting kernel-related traits are shown in Table 4.

^c^ Flanking markers of the QTL.

^d^ Numerals before parentheses are estimated of the additive effects of the QTL. Positive values indicate that SX828 alleles increase the TKW. Negative values indicate that KN2007 alleles increase TKW. E and numerals in parentheses indicate the environment in which the QTL was detected and the percentage of phenotypic variance explained by the additive effects of the mapped QTL, respectively. A minus sign, ‘‘-’’, or a plus sign, ‘‘+’’, following the parentheses denotes the additive effect of a conditional QTL, in absolute values, that reduces or increases more than 10 % compared to the corresponding unconditional QTL, respectively. An equal sign, ‘‘=’’, is placed after the parentheses to denote a conditional QTL with an equal additive effect to that of the unconditional.
